# The dialogue dilemma: the role of patient-clinician communication for low-income people who smoke and manage multiple conditions

**DOI:** 10.3389/fmed.2025.1567725

**Published:** 2025-04-28

**Authors:** Monique T. Cano, Michael R. Lindstrom, Ricardo F. Muñoz

**Affiliations:** ^1^Department of Psychiatry, Yale University School of Medicine, New Haven, CT, United States; ^2^Institute for International Internet Interventions for Health (i4Health), Palo Alto University, Palo Alto, CA, United States; ^3^Department of Psychiatry and Behavioral Sciences, University of California, San Francisco, San Francisco, CA, United States; ^4^School of Mathematical and Statistical Sciences, University of Texas Rio Grande Valley, Edinburg, TX, United States

**Keywords:** patient-clinician communication, tobacco use, low-income adults, multimorbidity, self-rated health, culturally informed care, patient-centered care, health disparities

## Abstract

**Introduction:**

Adults from low-income backgrounds who smoke face significant health disparities related to tobacco use, often at disproportionately high rates. These individuals are more likely to endure multiple mental and physical (MP) health conditions, which can negatively influence their self-rated health (SRH). The quality and effectiveness of patient-clinician communication (PCC) can influence how patients perceive their own health. Understanding how PCC influences SRH among low-income adults who smoke and suffer from multiple MP conditions is essential for clinical care as multimorbidity is on the rise. This study examines how PCC may influence the health perceptions of low-income adults who smoke and have varying MP conditions.

**Methods:**

Low-income adults who smoke (*N* = 58) were recruited from the San Francisco Health Network (SFHN) and were assessed for number of MP conditions, PCC, and SRH. A moderation analysis was performed to examine whether PCC moderated relations between MP conditions and SRH. Follow-up analyses were conducted to examine differences and relationships among variables. In planned exploratory analysis, all possible choices for moderator-independent-dependent-variable selections to explore the best model fit were conducted.

**Results:**

The results revealed that PCC moderated the association between MP conditions (*p* < 0.05) and SRH. In follow-up analyses, number of MP conditions predicted poorer SRH for low-income smokers who experienced low (*p* < 0.001) and average (*p* < 0.01) levels of PPC but not high levels of PCC. In planned exploratory analysis, based on the Akaike Information Criterion, a quantitative basis for considering SRH as the dependent variable was established.

**Conclusion:**

The intersection of tobacco-related disparities among low-income adults who smoke and manage multiple MP conditions is complex. Among this vulnerable population, poor and average PCC adversely influences how patients perceive their own health. Results highlight the importance of quality and effective communication between patients and providers. A culturally informed patient-centered approach to care may improve PCC as it encourages collaborative, individually tailored treatment that empowers patients to actively participate in their own health care.

## 1 Introduction

Smoking remains a modifiable health risk that affects every organ in the body and is the leading cause of global preventable death and preventable disease (1, 2). Health disparities are directly linked to tobacco disparities (3, 4) and low-income people who smoke suffer from multiple social, medical, and psychological needs at disproportionate rates compared to those from higher income strata (5, 6). Like tobacco disparities, multimorbidity—the coexistence of two or more chronic conditions which can include a physical non-communicable disease of long duration, a mental health condition of long duration, and/or an infectious disease of long duration (7, 8)—is also linked to health disparities (8, 9). Research has demonstrated that individuals belonging to the lowest level of income are four times more likely to experience multimorbidity compared with the highest level of income (10) and smoking has been found to be one of the primary predictors of multimorbidity (11–13).

Self-rated health (SRH) is a brief, single proxy measure of overall health and is the most widely used measure of health in medical, social, and behavioral science research that utilizes survey data (14, 15). It has been widely validated and is a critical indicator and strong independent predictor of morbidity and mortality even after adjusting for covariates including demographic, mental health, physiological, and behavioral risk factors (16, 17). As such, individuals with poor SRH tend to be in mental, physical, and/or social distress (18). Individuals who indicate poorer self-rated health tend to suffer disproportionately from health disparities (19) and the cycle of poor health within low-income populations has been extensively documented (20, 21). Behavioral risk factors, such as smoking, have been identified as key components that impact SRH (22, 23). Prior research demonstrates that frequent smoking is associated with over twice the odds of reporting poor SRH (22). Likewise, SRH is influenced by multimorbidity which may be due to the physiological consequences and psychological distress of managing multiple conditions (8, 24). Managing multiple chronic conditions in turn affects daily functioning and consequently individuals with multimorbidity tend to experience a decline in their quality of life (25). Thus, multimorbidity has been found to be negatively associated with SRH (26, 27).

Patient-clinician communication (PCC) plays an integral role in the management of mental and physical conditions (28). While there is no general consensus regarding the operational definition of PCC (29), in the current study PCC is defined as the extent to which a patient prepares for appointments, seeks clarification through active questioning, and engages in personal disclosure to enhance understanding and treatment outcomes. This definition highlights both the patient’s responsibility to actively engage in their care while also recognizing the clinician’s responsibility to foster trust (e.g., creating space for patients to share relevant personal concerns that may impact their health and/or treatment) and open communication. Quality PCC has been associated with greater satisfaction of care, medication adherence, and better health outcomes (28, 30–32). PCC is particularly important with low-income and underrepresented populations who tend to suffer disproportionately from health disparities (33, 34). Some factors that may deleteriously impact PCC in low-income and underrepresented populations include unmet social needs (e.g., food and housing insecurity), lower education levels, acculturation level, discrimination, immigrant status, language barriers (e.g., limited English proficiency), and low health literacy (28, 35). These patient-level factors impact PCC on various levels. For example, patients with limited English proficiency may experience challenges in understanding and adhering to medical advice from providers which could result in further delays in receiving appropriate medical care and medical errors (36). Similarly, patients with low health literacy have difficulty disclosing health related information due to limited health knowledge and have difficulty following treatment instructions prescribed to them by their providers leading to poorer health outcomes (37–39).

In addition to the aforementioned factors, low-income people who smoke who are at a higher risk for multimorbidity also have a greater risk for experiencing adverse health outcomes, receiving conflicting treatment recommendations due to having multiple clinicians and conditions, and experience greater self-management burdens (8, 40, 41). Thus, these patients have a greater need for clear and effective PCC. Barriers to effective PCC such as limited English proficiency and low health literacy have been linked to poorer SRH even after adjusting for covariates (42). SRH is linked to communication inequalities—differences in socioeconomic status (SES), race, gender that impact access and ability to take advantage of the information given by providers—that are impacted by the social determinants of health with health information avoidance mediating this relationship (43). Conversely, quality PCC is associated with better SRH and better patient-clinician relationships (31). Quality PCC is associated with higher treatment satisfaction, medication adherence, better self-management, reducing stigma, and higher subjective decision quality, all of which may positively impact SRH (30, 32, 44–46).

Low-income people who smoke experience health disparities and are more likely to suffer from multimorbidity, both of which adversely impact patient SRH (12, 13, 33, 34). Similarly, depending on its quality and effectiveness, PCC can impact SRH adversely or positively (28, 31, 32, 45). Although existing literature has examined various elements of PCC across different healthcare settings, there is a gap in the literature regarding how PCC influences SRH among low-income people who smoke and who suffer from multiple chronic conditions. Understanding this interaction is essential as multimorbidity has proven to be an escalating global issue with considerable clinical implications, especially for those who are socioeconomically deprived and smoke (8). Therefore, the primary aim of the current study examines how the number of mental and physical (MP) conditions and PCC interact and relate to SRH. We hypothesized that PCC would moderate the relationship between number of MP and SRH, where greater PCC would result in better SRH. In planned exploratory analysis, we examined all possible choices for moderator-independent-dependent-variable selections to explore the best model fit.

## 2 Methods

### 2.1 Participants and procedures

The current study is based on secondary analyses from an outcome study that was initially developed to explore differences between low-income people who smoke with and without chronic illnesses in terms of nicotine dependence, depression, anxiety, and smoking abstinence self-efficacy. Participants were recruited from primary care clinics within the San Francisco Health Network (SFHN) and other sites in the San Francisco Bay Area, that primarily serve low-income individuals. Recruitment flyers were posted in waiting rooms and other locations with the approval of each of these sites. Staff at various recruitment sites were also provided with flyers to inform prospective participants about the study. Eligible participants were English-speaking, low-income, as defined by the poverty threshold for San Francisco Bay Area residents (47), adults (aged ≥18 years) who smoke and have thought about or intended to quit smoking within 30 days. All procedures were approved by the University of California, San Francisco and Palo Alto University.

### 2.2 Measures

#### 2.2.1 Demographic questionnaire

Participants reported their age, race, ethnicity, gender, marital status, employment status, total household income, and years of education.

#### 2.2.2 Number of mental and physical (MP) conditions

Four different measures were used to assess for mental and physical conditions. To assess for mental health conditions, participants completed the Generalized Anxiety Disorder – 7 (GAD-7) (48) and the Patient Health Questionnaire (PHQ-9) (49). Cut off scores for the GAD-7 and PHQ-9 were ≥8 and ≥10, respectively, to categorize participants as screening positive for a diagnosable generalized anxiety disorder and/or major depressive episode (50). Nicotine dependence was conceptualized as a mental health condition for this study and to assess for nicotine dependence, participants completed the Fagerström Test of Nicotine Dependence (FTND). A cut-off score of 6 was utilized to determine whether a participant was dependent on nicotine (51).

To assess for chronic physical conditions, a self-report questionnaire asking for the presence or absence of 22 chronic illnesses over the past year was administered, modeled after the physical health self-report module described by Atwoli et al. (52). The list of chronic physiological conditions included: high blood pressure, high cholesterol, heart attack, stroke, heart disease, asthma, seasonal allergies, other lung disease, diabetes, thyroid condition, osteoporosis, acid reflux, ulcer, obesity, arthritis, chronic pain, frequent headaches, neurological disease, epilepsy, cancer, kidney disease, and other, to be specified by the participant.

#### 2.2.3 Self-rated health

Self-rated health was measured by a well-established single item developed by the Stanford Patient Education Research Center, which has been widely used in diverse samples to assess for perceived general health status (42, 53, 54). The item asked, “In general, would you say your health is…” There were five response options: 1 (“Excellent”), 2 (“Very good”), 3 (“Good”), 4 (“Fair”) and 5 (“Poor”). Higher scores on this scale signify the patient perceives their health as worse.

#### 2.2.4 Patient-clinician communication

Patient-clinician communication was measured by a three-item questionnaire developed by the Stanford Patient Education Research Center (54). The three items begin with, “When you visit your doctor, how often do you do the following” and include the following: (1) “Prepare a list of questions for your doctor…,” (2) “Ask questions about the things you want to know and things you do not understand about your treatment…,” and (3) “Discuss any personal problems that may be related to your illness…” Responses are coded on a 6-point Likert scale ranging from 0 “Never” to 5 “Always.” The total score is the mean of the three items. Higher scores indicate better communication.

### 2.3 Statistical analyses

#### 2.3.1 Power estimates

An *a priori* power analysis was performed for sample size estimation using G*Power (55) version 3.1.9.2. This study was designed for adequate power for a moderated regression that examined the interaction between two predictors (number of MP and PCC). Power calculations were conducted based on a 0.05 alpha level and 80% power (1-*β*). These calculations suggested that for a moderated regression, total sample size of 25 to 55 is required to detect a medium to large effect size via Cohen’s ƒ^2^ (0.15 ≤ effect size ≥ 0.35). Given these power approximations, we expected our study sample of 58 to be appropriately powered to detect the hypothesized effects.

#### 2.3.2 Data processing

All records for which a patient had missing data, be that a missing yes/no for a health condition or a missed question on an instrument such as PCC were removed; a total of 6 participants were removed.

#### 2.3.3 Descriptive and statistical model analyses

Analyses for the primary aim were performed using IBM SPSS Statistics for Macintosh Version 29 (IBM Corp., Armonk, N.Y., USA). Prior to analyses, bivariate correlations were examined among predictor variables to test for multicollinearity and dependent variable normality was confirmed. To examine the interplay between number of MP conditions, PCC, and SRH, a moderation analysis was performed using Hayes PROCESS Model 1 (56). The model included SRH as the criterion, with number of MP conditions, PCC, and number of MP conditions-by-PCC interaction term as fixed effects. We included age (24–70 years old), gender, race, and years of education as covariates in the model. To clarify the direction of a significant interaction, we performed follow-up tests that examined associations between number of chronic conditions and SRH at low, average, and high levels of PCC, where low and high represent 1 standard deviation below and above the group mean, respectively.

#### 2.3.4 Moderation model comparisons for exploratory analyses

Under moderation (57), the dependent variable Y, the independent variable X, and the moderator M are related via:

$$Y=\left(\alpha_{1}+\beta_{1}M\right)+\left(\alpha_{2}+\beta_{2}M\right)X$$

where $\alpha_{1},\beta_{1},\alpha_{2},\mathrm{and}\;\beta_{2}$ are regression variables. Thus, at each fixed value of the moderator M, Y is a linear function of X, but that linear relationship can change with M. We restrict ourselves to the case that the errors are normally distributed. Among the models that are normally distributed, we compare them based on the Akaike Information Criterion (58) to determine the best model.

The models for planned exploratory analyses were run in Python 3.9.12. Parameters were computed via least squares using the *Numpy* library (59). Normality was tested for with the Shapiro-Wilks Test from the *SciPy* library (60) at a *p*-value of 0.05.

## 3 Results

### 3.1 Participant characteristics

The total sample consisted of 58 low-income primary care patients who smoke. The age ranged between 24 and 70 years (44.97 ± 10.69). The mean years of education attainment was 12.05 ± 2.82 years of schooling. Most participants identified as cisgender males (*n* = 37; 63.8%) and about one third identified as cisgender females (*n* = 20; 34.5%), with one participant identifying as transgender. Most participants identified as not Latinx/Hispanic (*n* = 35; 60.3%), Non-Hispanic White (*n* = 20; 34.5%), and African American/Black (*n* = 18; 31.0%). The remainder of the participants identified as Asian (*n* = 1; 1.7%), Mestizo (*n* = 2; 3.4%), Native American/Alaskan Native (*n* = 3; 5.2%), Native Hawaiian/Pacific Islander (*n* = 2; 3.4%), Biracial (*n* = 11; 19.0%), or an unknown or unreported racial identity (*n* = 1; 1.7%). Most participants reported that they were single/never married (*n* = 35; 60.3%), followed by married or in a domestic partnership (*n* = 10; 17.2%), separated (*n* = 3; 5.2%), divorced (*n* = 7; 12.1%), and widowed (*n* = 3; 5.2%). Most participants reported being unemployed (*n* = 42; 72.4%) and reported a total household income of less than $20,000 per year (*n* = 47; 81.0%). Many participants reported having three or more clinical conditions within the past year (*n* = 26; 44.8%). Characteristics of the sample are summarized in Table 1.

**Table 1:** Characteristics of the sample.

	Total *N* = 58
Demographics	
Gender *n* (%)	
Male	37 (63.8)
Female	20 (34.5)
Transgender	1 (1.7)
Age (mean, SD)	44.97 (10.69)
Education (mean, SD)	12.05 (2.82)
Race *n* (%)	
Non-Hispanic White	20 (34.5)
African American/Black	18 (31.0)
Biracial	11 (19)
Native American/Alaskan Native	3 (5.2)
Mestizo	2 (3.4)
Native Hawaiian/Pacific Islander	2 (3.4)
Asian	1 (1.7)
Unknown/Not reported	1 (1.7)
Ethnicity *n* (%)	
Not Latinx/Hispanic	35 (60.3)
Latinx/Hispanic	14 (24.1)
Unknown/Not reported	9 (15.5)
Marital Status *n* (%)	
Single (never married)	35 (60.3)
Married, or in a domestic partnership	10 (17.2)
Separated	3 (5.2)
Divorced	7 (12.1)
Widowed	3 (5.2)
Employment Status *n* (%)	
Full-time	6 (10.3)
Part-time	2 (3.4)
Retired	2 (3.4)
Student	6 (10.3)
Unemployed	42 (72.4)
Household Income *n* (%)	
<$20,000	47 (81.0)
$20,000–$34,999	3 (5.2)
$35,000–$49,999	3 (5.2)
$50,000–$74,999	2 (3.4)
$75,000–$99,000	2 (3.4)
Unknown/Not reported	1 (1.7)
Number of mental and physical conditions *n* (%)	
0 conditions	11 (19.0)
1 condition	12 (20.7)
2 conditions	9 (15.5)
3 + conditions	26 (44.8)

### 3.2 Moderation analysis

#### 3.2.1 Interaction between number of MP conditions and PCC on SRH

**Figure 1:**
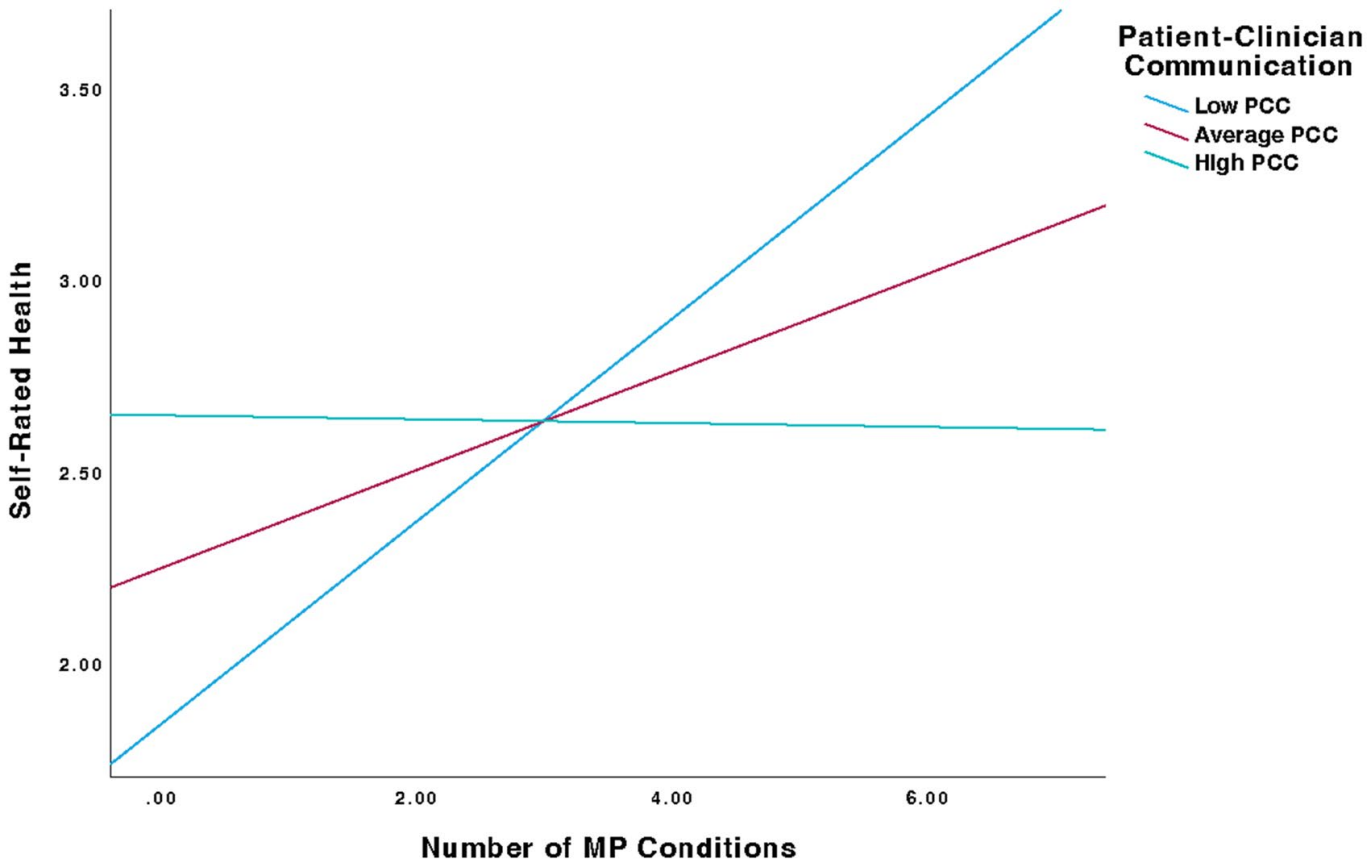
Moderation analysis illustrating the interaction between number of MP conditions (X) and patient-clinician communication (M) in predicting self-rated health (Y); results indicate a significant interaction effect (*p* < .05).

**Figure 2:**
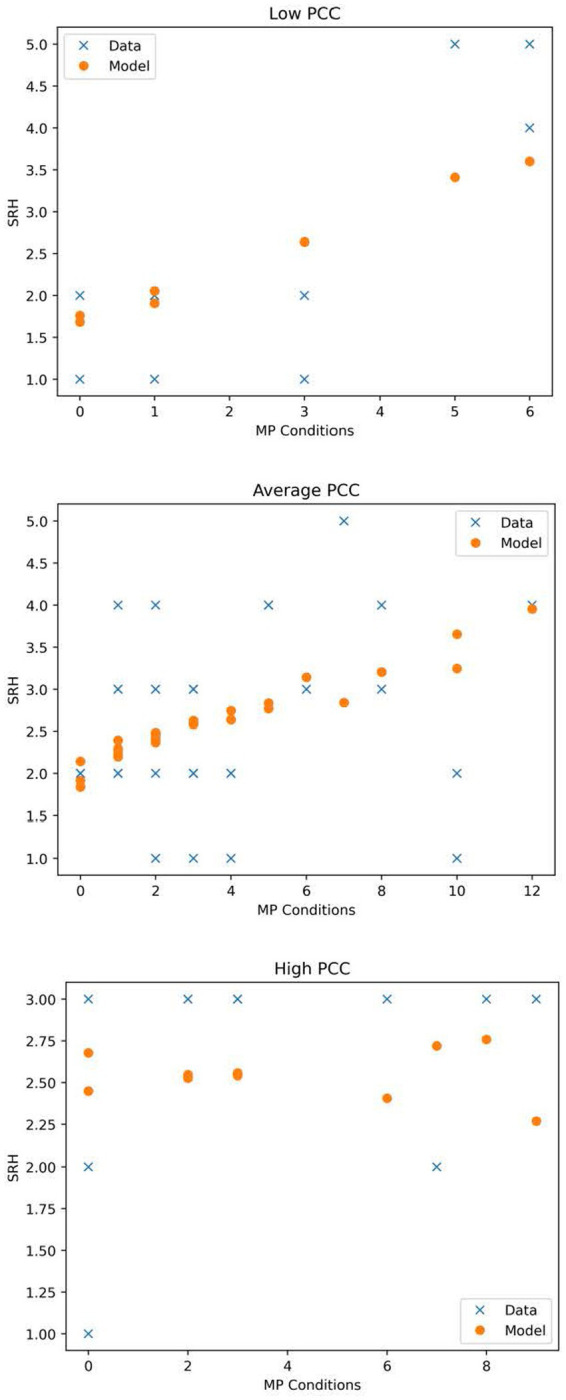
Expanded visualization of the interaction between number of MP conditions and patient-clinician communication in predicting self-rated health. This figure depicts both the moderation model (see equation) and raw data for patients with low (1 or more standard deviations below the mean), average (within one standard deviation of the mean), and high (1 or more standard deviations above the mean) levels of patient-clinician communication.

Results indicate that the overall moderation model was significant (*p* < 0.01) and a significant number of MP conditions-by-PCC interaction for SRH was observed [β = −0.09, (95% CI, −0.17 to −0.02), *p* < 0.05]. Further interrogation of this interaction revealed that number of MP conditions predicted SRH at low and average levels of PCC [Low: β = 0.26, (95% CI, 0.10–0.43), *p* < 0.001]; [Average: β = 0.13, (95% CI, 0.03–0.23), *p* < 0.01] but not high levels of PCC [High: β = −0.01, (95% CI, −0.13 – 0.12), *p* = 0.93]. Estimated marginal means of linear trends for this model can be found in Table 2 and a graphical representation is located in Figure 1; for more in-depth perspective of the moderation, that includes the moderation predictions and the raw data, see Figure 2. Results also indicated that covariates had no significant influence on the IV.

**Table 2:** Estimated marginal means of linear trends for moderation model.

	B (SE)	95% confidence interval		*p*-value
		Lower	Upper	
PCC ~ SRH				
−1 SD PCC (1.10)	0.26 (0.08)	0.10	0.43	0.001
Mean PCC (2.53)	0.13 (0.05)	0.03	0.23	0.01
+1 SD PCC (3.95)	−0.01 (0.06)	−0.13	0.12	0.93

### 3.3 Exploratory analyses

A summary of the different moderation models can be found in Table 3. The best models due to the lowest AIC-value have SRH as the dependent variable with PCC and number of MP conditions both being equally good moderators/independent variables. This is to be expected because, based on the form of the model, X and M can be switched and the model is the same. The AICs were not reported with number of MP conditions being a dependent variable because the errors were not normally distributed. The data thereby provide a quantitative basis for making SRH a dependent variable, with either PCC or number of MP conditions being the moderator.

**Table 3:** Summary of continuous moderation models.

Moderator	Independent	Dependent	Normal Errors	AIC
PCC	MP conditions	SRH	Yes	70.2
MP conditions	PCC	SRH	Yes	70.2
SRH	MP conditions	PCC	Yes	112.0
MP conditions	SRH	PCC	Yes	112.0
SRH	PCC	MP conditions	No	N/A
PCC	SRH	MP conditions	No	N/A

## 4 Discussion

The results of this study magnify the significance of culturally tailoring interventions that aim to improve PCC for health disparity and minority populations. The management of multiple conditions, each with their own complex set of treatment regimens prescribed by different providers, is challenging (8). Clear and effective PCC is especially important for low-income people who smoke as they are significantly impacted by pervasive social inequities in health and are more likely to suffer from multimorbidity and its associated adverse health outcomes (10, 11, 13, 28, 30). Our primary findings indicate that PCC plays a role in how low-income people who smoke and have multiple health conditions perceive their overall health. In a moderation model, we observed an interaction between number of MP conditions and PCC on SRH, where the association between number of MP conditions and SRH differed according to low, average, or high levels of PCC. Specifically, for low-income primary care patients who indicated low (i.e., 1 standard deviation below the mean) or average PCC, number of MP conditions was associated with poorer SRH, whereas number of MP conditions and SRH were unrelated among patients who indicated high PCC. Additionally, findings from exploratory analyses indicated that the direction of the conceptual model represented in the study holds strong quantitatively as well. The exploratory findings suggest that an interaction between the number of MP conditions and the quality of PCC does indeed impact SRH.

These findings suggest that poor and average PCC may adversely influence how low-income people who smoke and have multiple conditions may perceive their own health. This is consistent with a study that found a strong association between the patient-provider relationship and SRH among vulnerable patients; additionally, the study found that good patient-provider relationships were also associated with quality of care such as patient satisfaction and health outcomes (31). Previous studies have drawn similar conclusions and have found that PCC directly impacts health outcomes (61). For example, in a recent study, patients with uncontrolled blood pressure were more likely to improve their hypertensive outcomes when being treated by physicians who underwent a communication skills training program (62). Likewise, in another study, Black patients who report high levels of communication with their physicians were more likely to adhere to their medication regimen (30). Higher levels of PCC may make a significant difference in a patient’s quality of life and impact future morbidity and mortality given its direct influence over health outcomes. Interestingly, the findings also indicated that MP conditions and SRH were unrelated among patients who indicated high PCC. While current research indicates that effective PCC is often associated with positive health outcomes, studies have also demonstrated that PCC may not always have a significant impact on SRH (63). Multiple factors such as organizational and heath care system culture, being proactive as a patient, and health literacy may significantly contribute to SRH among those with multiple chronic illnesses despite effective PCC (31, 64).

Quality PCC is particularly important as it may serve as a function to reduce or maintain health disparities among vulnerable populations such as low-income people who smoke. PCC is influenced by SES in that providers tend to give fewer positive socio-emotional responses, engage in a directive consultation style, and provide less information and direction to lower SES patients while higher SES patients tend to experience a more egalitarian model of patient-provider communication (65). Among low-income patients, having a more egalitarian doctor-patient relationship (e.g., patient centered care) has been found to have better ratings of SRH whereas more traditional paternalistic doctor-patient relationship has been associated with worse SRH (66). It is important to note that socioeconomic status (SES) is merely one aspect of a patient’s multiple intersecting identities that may influence a provider’s communication style; ineffective communication that fails to account for the multiple cultural nuances of one’s identity may prove detrimental during a significant clinical encounter.

A patient centered approach to care is one such method that may improve PCC among low-income people who smoke and lead to more culturally informed and context-based interventions during clinical encounters. Health care providers who engage in patient-centered care are encouraged to take a collaborative approach in which patients and providers design personalized care together; this provides patients with high-quality, individually tailored care that may improve the efficiency and effectiveness of health care systems (67). Patient centered care has been found to improve provider behaviors—e.g., interpersonal skills, history taking, counseling—during clinical encounters with minoritized patients (68) and empowers patients to engage in shared decision making in their own health care (69).

Effective and productive communication during clinical encounters is also influenced by a patients’ level of active participation (70). While there is a vast literature focused on improving provider communication in clinical encounters, research that centers on training patients to interact more productively with their providers is largely underdeveloped (70). Patients who are active communicators with their providers tend to state their treatment preferences more frequently, experience improved psychological wellbeing, and are more likely to check for understanding to confirm the factors and issues being discussed during clinical encounters (71–73). With support and resources, encouraging low-income patients to improve their communication skills can be both achievable and impactful. Teaching and empowering underserved populations to become effective communicators with their health care providers has been found to improve health outcomes (70), which may also play a role in alleviating health disparities. Likewise, educating patients on how to engage more effectively with their providers may improve health literacy, build confidence in navigating the healthcare system, and empower them to demonstrate more respectful and assertive communication (70, 72, 74, 75). By providing patients with skills to prepare for appointments, seek clarification through active questioning, and engage in open communication about their concerns, we can foster stronger, more collaborative relationships, ultimately leading to better health outcomes.

This study has several limitations. First, the cross-sectional nature of the research design limits our ability to make causal interpretations of the results. Although we were able to gather meaningful insights on when PCC may impact SRH among low-income people who smoke, we did not measure changes through time. Second, although adequately powered for the analyses that were run, the study had a relatively small sample size which limited our ability to run more robust analyses. Third, given that the sample consists of low-income people who smoke who were recruited from an urban area of the United States, we are unable to generalize to other low-income populations who reside in rural areas or areas outside of the United States. In addition, given the voluntary nature of the study, the results presented cannot be generalized to individuals who chose not to participate. Fourth, this study relied on self-report data; the mental and physical conditions were not objectively measured by providers who could assess and draw conclusions for formal diagnoses. Fifth, the three item PCC measure utilized in this study focuses exclusively on the patient perspective of PCC, which may result in an oversimplified view of a complex relationship between patient and provider; however, it is important to note that the provider bears the responsibility to prioritize patient values, needs, and preferences throughout the provision of care (64). Sixth, the authors recognize that there are other unmeasured factors that may influence the moderation model reported in this study such as different aspects of the social determinants of health (e.g., discrimination, access to care, health literacy) and medical mistrust. Despite these limitations however, the study team was able to draw meaningful conclusions on the perceptions of low-income people who smoke, particularly in the context of their varying levels of communication quality with healthcare providers. Furthermore, this study provides additional insights into a population that is greatly impacted by the social determinants of health.

Future research in this area is warranted. Future intervention research focused on provider-patient communication may consider a motivational interviewing (MI) approach which takes a collaborative, acceptance based, compassionate, and empowering approach to care (76). Additionally, it may be important for providers to receive training on how to conceptualize a patient’s readiness for change while prioritizing patient well-being. Providing training to healthcare providers on person-centered, collaborative treatment approaches in low-income clinical settings may improve SRH. Future studies may consider a longitudinal approach to better understand how SRH may change over time in low-income people who smoke based on changes in PCC quality. Finally, future investigations focused on teaching patients how to interact more productively—e.g., interpersonal effectiveness skills training—with their providers may improve PCC.

## Data Availability

The datasets presented in this article are not readily available because of confidentiality regarding the involvement of a vulnerable population. Requests to access the datasets should be directed to monique.cano@yale.edu.
